# Construction of PVDF/BTO Fibers with High Piezoelectricity for Self-Powered Flexible Pressure Sensing

**DOI:** 10.3390/polym17223043

**Published:** 2025-11-17

**Authors:** Zao Liu, Hongjian Zhang

**Affiliations:** 1 Cooperative Innovation Center of Industrial Fermentation (Ministry of Education & Hubei Province), Key Laboratory of Fermentation Engineering (Ministry of Education), Hubei Key Laboratory of Industrial Microbiology, Hubei University of Technology, Wuhan 430068, China; 2 School of Mechanical Science & Engineering, Huazhong University of Science and Technology, Wuhan 430074, China

**Keywords:** piezoelectric, energy harvesting, pressure sensor, power density, composites

## Abstract

As demanded by the rapid development of wearable electronics, flexible pressure sensors have attracted more and more attention worldwide. However, the performance of pressure sensors based on the piezoelectric effect has not been qualified for practical application. In this work, we designed and prepared PVDF/BTO fibers by an electrospinning approach and constructed pressure sensors for various human motion detection purposes. The performance is dramatically enhanced due to the high piezoelectric coefficient of BTO nanoparticles and the utilization of PVDF matrix with a high β content. The optimized pressure sensor presents a high power density of 27.5 nW cm^−2^ at 10 N load stress and remains stable even after 3000 cycles. For human action application, the maximum output voltage is up to 20 V. The optimum β content of the PVDF matrix is up to 72.9%, which in turn contributes to the high energy-harvesting capability of the designed composites. The prepared PVDF/BTO composites show benign capability for sensing various human actions. This work shapes a new strategy for high-performance pressure sensors based on piezoelectric composites.

## 1. Introduction

In recent years, pressure sensors have attracted more and more attention due to the rapid development of the Internet of Things (IoTs) and human–machine interfaces [1,2,3,4,5]. Currently, there are four types of pressure sensors, including piezoelectric, triboelectric, capacitive, and resistive [6,7]. Among them, piezoelectric-type pressure sensors present the typical advantages of being self-powered, low cost, long-term stability, etc., thus becoming the mainstream research topic in this field. Especially, the self-powered characteristic helps to extend the usage life or diminish the utilization of electrochemical batteries for powering pressure sensors, which is rather important for practical applications [4,8]. For human use, the Young’s modulus of piezoelectric ceramics is too high, while the performance of pure piezoelectric polymer has not been qualified. Thus, composites made of polymer matrix and piezoelectric fillers represent good candidates, which harness the advantages of the high piezoelectricity of ceramics and benign flexibility of polymer matrix simultaneously [9,10]. The development of piezoelectric nanocomposites is rapidly advancing on multiple fronts, propelled by both the discovery of novel polar polymer systems with unique electromechanical responses and the implementation of data-driven approaches, such as high-throughput phase-field simulation and machine learning, for their rational design and optimization [11,12].

PVDF is a good polymer matrix due to its high piezoelectric coefficient, chemical stability, easy processibility, etc. PVDF includes five different crystalline phases (α, β, γ, δ, and ε) [13,14]. Among them, α and β phases are the phases with piezoelectricity, and β phase is better [15,16]. So the pursuit of a high β phase content in PVDF is important for electrical performance optimization. For human applications, for example, flexible electronics and implanted devices, we should select lead-free piezoelectric ceramics due to their biocompatibility. As a typical lead-free piezoelectric ceramic, BaTiO_3_ (abbreviated as BTO) possesses high piezoelectricity and easy preparation characteristics [17,18]. While conventional PVDF-based composites incorporating zero-dimensional piezoelectric particles have shown promise, their performance remains constrained by fundamental material limitations. The tendency for nanoparticle agglomeration creates heterogeneous piezoelectric responses, while the discontinuous interface between filler and matrix leads to inefficient mechanical stress transfer, resulting in substantial energy dissipation. The electrospinning process is a widely used approach for preparing flexible devices, due to its high construability, compatibility for large-scale and commercial production, etc. [19,20]. To date, a variety of studies have been conducted based on the electrospinning process for flexible pressure sensing. As reported, PVDF/BaTiO_3_ nanocomposites prepared via the electrospinning process were able to serve as energy harvesters and self-powered vibration sensors [21,22]. However, their performance has still not been qualified for practical applications.

In this work, we composited the PVDF matrix with BTO fillers by the electrospinning process. By modifying the BTO filler content and preparation parameters, we designed and prepared a series of high-performance self-powered pressure sensors. This work distinguishes itself from conventional film-based PVDF/BTO composites through its unique electrospun fibrous architecture. This structural advantage endows the material with superior flexibility and enhanced stress concentration capability, which are critical for high-performance wearable sensing applications. A variety of characterizations were conducted to investigate the structure–property correlation in PVDF/BTO composites. The piezoelectric energy-harvesting capability is up to 27.5 nW cm^−2^ when the BTO content is 5 wt%. The maximum output voltage is up to 20 V when they are acted on by the foot, which is comparable with the results in previous studies or even better. The long-term stability is also confirmed by the 3000 cycles test, confirming the benign integration of two phases (PVDF matrix and BTO fillers). We attribute the enhanced performance to the high β content of the PVDF matrix and the introduction of high-piezoelectricity BTO fillers. A variety of applications were realized by using the prepared self-powered pressure sensors, which further demonstrates the high possibility for commercial usage.

## 2. Experimental Section

### 2.1. Materials

Aluminum foil, ethyl alcohol (C_2_H_5_OH), barium carbonate (BaCO_3_, purity > 99.5%), and titanium dioxide (TiO_2_, purity > 99%) were supplied by Sinopharm Chemical Reagent Co., Ltd. (Shanghai, China). *N*-Dimethylformamide (C_3_H_7_NO, DMF) and acetone (CH_3_COCH_3_) were supplied by Aladin Co. (Shanghai, China). The polymer matrix of polyvinylidene fluoride ((CH_2_CF_2_)*_n_*, PVDF, MW ∼ 543,000) was obtained from Piezotech Co. (Paris, France). All the above chemicals were used without further purification.

### 2.2. Preparation of BTO Fillers and PVDF/BTO Fibers

The solid-state method was used to prepare BTO particles. BTO particles were synthesized via a conventional solid-state reaction method. Briefly, high-purity barium carbonate (BaCO_3_) and titanium dioxide (TiO_2_) powders were mixed in a stoichiometric molar ratio of 1:1 and homogeneously blended using a planetary ball mill operating at 300 rpm for 24 h. The resulting mixture was subsequently calcined in a high-temperature furnace at 1400 °C for 4 h under an air atmosphere to facilitate complete perovskite crystallization. PVDF (2 g) was fully dissolved in DMF solvent (5 mL) and magnetically stirred at 70 °C for 0.5 h to form a clarified solution. Then, distinct contents of BTO fillers were introduced in the PVDF solution. We utilized the stirring process (50 °C for 5 h) to form a homogeneous solution. The prepared electrospinning solution was pumped into a plastic syringe. In this work, we controlled the feed rate of the plastic syringe at 0.1 mm/min for rational fiber diameter. The electrospinning voltage was set at 10 kV and not changed throughout the process. The specific parameters were as follows: A needle with an inner diameter of 1.84 mm was used. The tip-to-collector distance was set to 15 cm, and the electrospinning process was carried out under ambient conditions with a temperature of 26 °C and a relative humidity of 30%. Furthermore, the rotation speed of the fiber collector was controlled at 2800 rpm. Finally, the PVDF/BTO fibers were completely dried in a 70 °C vacuum oven for 10 h.

### 2.3. Characterizations

The crystal structure was characterized by X-ray diffraction patterns (XRD, Cu Kα radiation, λ = 1.54 Å, 40 kV, 20 mA, Rigaku Smartlab). Surface morphologies were determined by field emission scanning electron microscopy (FE-SEM, JSM-7610F, Rigaku, Tokyo, Japan). Copper foils (thickness around 0.1 mm) were attached on two sides of the PVDF/BTO fibers to serve as electrodes for electrical performance characterization. The generated energy-harvesting and sensing signals were collected with a digital electrometer (Keithley 6517B, Keithley, Solon, OH, USA).

## 3. Results and Discussion

**Figure 1:**
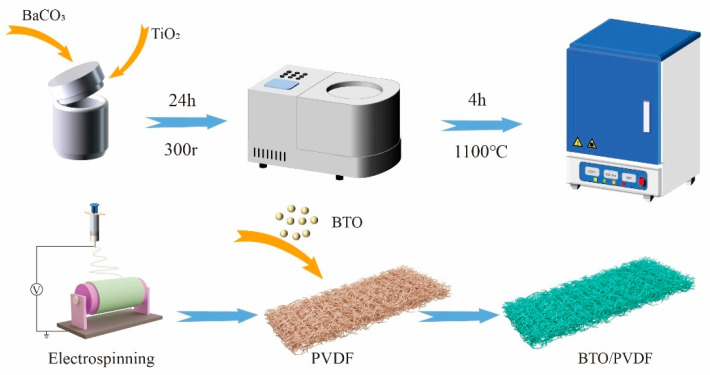
Schematic of the detailed preparation process of BTO fillers and electrospinning PVDF/BTO fibers.

**Figure 2:**
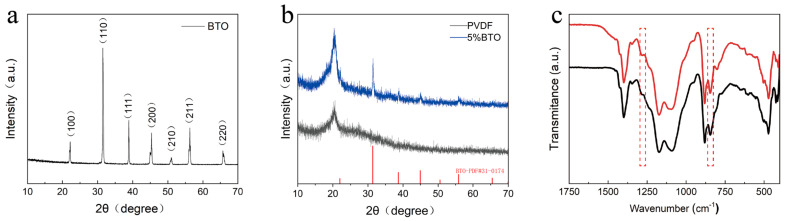
(**a**) XRD pattern of BTO fillers; (**b**) XRD patterns of PVDF and PVDF/BTO-5; (**c**) FTIR spectrum of pure PVDF PVDF/BTO-5.

Figure 1 shows the detailed preparation process of the BTO particles and PVDF/BTO composite fibers. The BTO particles were prepared by the traditional solid-state method, for which the sintering temperature was set at 1400 °C to guarantee the successful transition of the tetragonal phase. For BTO ceramics, the tetragonal phase is demanded for high piezoelectricity. Figure 2a shows the XRD pattern of BTO particles, in which we can see the obvious peak spilt at 45°. The peak spilt at 45° confirms that the BTO presents a tetragonal phase [23,24]. And no impurity could be observed in the XRD pattern, which confirms a pure phase of BTO fillers. The PVDF/BTO composites were prepared by an electrospinning process, in which the BTO fillers were fully wrapped in PVDF matrix. Figure 2b shows the XRD patterns of pure PVDF and PVDF/BTO-5. We can see the crystal peaks of PVDF and BTO simultaneously. The β phase in PVDF is confirmed by the peak at 20°, which corresponds to the diffraction from the (110) and (200) lattice planes of the β phase, while the typical peak of BTO is confirmed by the peak at 30° [25]. In this work, the BTO content was varied from 0 wt% to 7 wt%. The composites were named PVDF/BTO-0, PVDF/BTO-3, PVDF/BTO-5, and PVDF/BTO-7, respectively.

**Figure 3:**
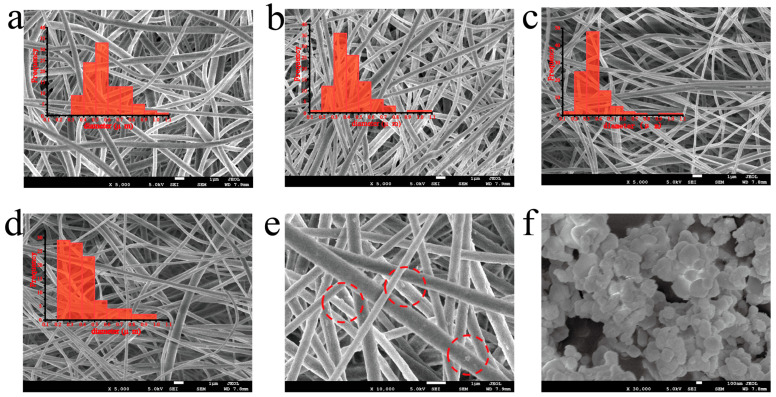
(**a**–**d**) SEM images of pure PVDF and PVDF/BTO-3, PVDF/BTO-5, and PVDF/BTO-7 composites; the insets show the diameter distribution of the electrospinning fibers. (**e**) Enlarged view of PVDF/BTO-5 composite to show the BTO fillers wrapped in PVDF matrix; (**f**) SEM image of BTO fillers.

The SEM images of electrospinning fibers with BTO content varying from 0 to 7 wt% are shown in Figure 3a–d. As can be seen, the BTO fillers are closely wrapped in the PVDF matrix and no aggregates could be observed. We can clearly see the BTO fillers in the PVDF matrix, as highlighted by the red circles (Figure 3e). The inset images present the diameter distribution of the fibers, calculated for more than 200 fibers. We can see the gradual increase in diameter, which is due to increased viscosity with BTO introduction. Due to the BTO content not varying too much, the diameter changed only slightly. The size of PVDF/BTO-5 is more concentrated around 0.4–0.5 μm. Furthermore, the BTO content influences the solution viscosity, which consequently modulates the fiber diameter during electrospinning. This change in fiber morphology alters the available surface area and the distribution of BTO particles, thereby affecting the stress transfer efficiency and ultimately governing the resultant electrical output. In this work, the size of the BTO fillers is around 100–200 nm, as shown by Figure 3f. As can be seen from the X-ray diffraction pattern (Figure 2b), the BTO fillers present a pure tetragonal phase with no impurity. From the FT-IR spectrum (Figure 2c), we can calculate the β phase content of various composite fibers.

The characteristic absorption bands of the β phase were characterized at 840 cm^−1^ and 1275 cm^−1^. Meanwhile, the characteristic absorption bands of the α phase were characterized at 763 cm^−1^. We can calculate the β phase content in the PVDF/BTO fibers by using the following equation [26,27].

*F*(*β*) = *X*_*β*_/(*X*_*α*_ + *X*_*β*_) = *A*_*β*_/(*K*_*β*_/*K*_*α*_)*A*_*α*_ + *A*_*β*_ (1)

In the equation, *A_α_* and *A_β_* represent the absorbance at 763 cm^−1^ and 840 cm^−1^, respectively, corresponding to the α and β phases of the PVDF matrix.

As the BTO fillers are introduced, the β phase content is increased, which is in agreement with previous studies. As we know, the introduction of conductive or inorganic fillers helps to improve the β phase content in PVDF matrix. The observed enhancement in β phase content is primarily attributed to the synergistic effect of strong electrostatic interactions and heterogeneous nucleation. The surface ions of BaTiO_3_ generate a local electric field, which promotes the alignment of PVDF molecular dipoles into the all-trans conformation characteristic of the β phase. Concurrently, the BaTiO_3_ nanoparticles act as efficient nucleating agents, reducing the energy barrier for the formation of the polar phase [8,16,19]. In this work, the highest β phase content is up to 72.9% in the PVDF/BTO-5 composite.

**Figure 4:**
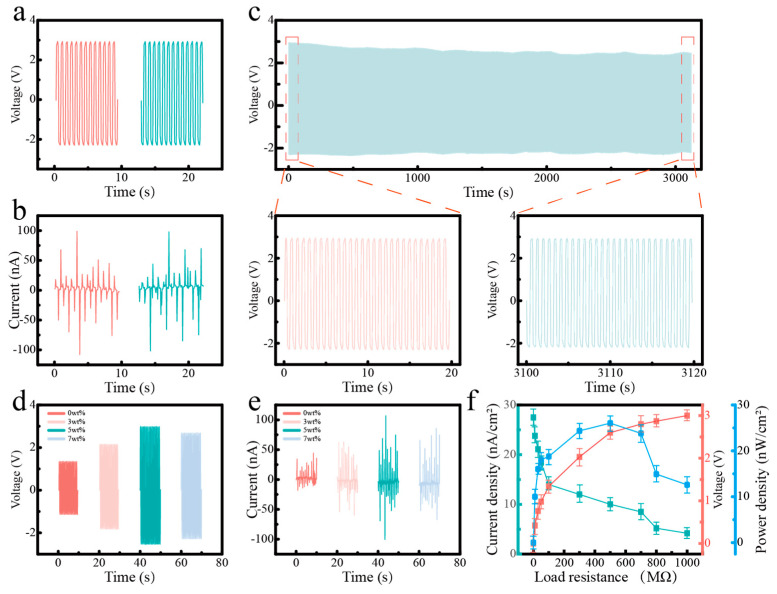
(**a**,**b**) Voltage and current of PVDF/BTO-5 composite with forward and reversed connections (load stress of 10 N); (**c**) 3000 cycles test and enlarged views; (**d**,**e**) output voltage and current of PVDF-based composites with different BTO contents; (**f**) the variations in voltage, current density, and power density of PVDF/BTO-5 composite with varying load resistance.

For the PVDF/BTO fibers, we investigated their energy-harvesting and sensing capability under different conditions. For the data in Figure 4, the stress was controlled at 10 N and the sample size was 1 × 1 cm^2^. For the data in Figure 4a,b, the forward and reversed connections were established to confirm that the signal was really from the piezoelectric effect [28,29,30]. The signal amplitude was almost the same with the forward and reversed connections. The output voltage was up to 3 V, and the current density was 100 nA cm^−2^. The long-term stability was confirmed by the 3000 cycles test, in which no noticeable degradation of performance could be detected. The 3000 cycles durability test was performed to simulate the continuous mechanical deformation experienced by the device when conformally attached to the human body during long-term motion. Thus, we concluded that the prepared PVDF/BTO composites are suitable for practical applications. With different contents of BTO fillers, the composites present distinct performance [31,32,33]. When the doping content is 5 wt%, the performance is the highest. Upon introducing BTO fillers in PVDF matrix, the output is increased with increasing BTO content, which is due to the high piezoelectricity of BTO fillers. For PVDF/BTO-7, the performance is lower than that of PVDF/BTO-5, which is due to the aggregation of excessive BTO fillers in the PVDF matrix, which in turn degrades the output. In detail, the performance variation with BTO content can be attributed to the evolution of the filler distribution within the fiber matrix. At low concentrations (<5 wt%), while the BTO nanoparticles are well-dispersed, the number of active piezoelectric units is insufficient to generate a strong composite response. In contrast, at high concentrations (>5 wt%), the excessive filler loading leads to nanoparticle agglomeration, which not only reduces the effective interfacial area for stress transfer but also creates localized defects that compromise both mechanical integrity and piezoelectric performance. The 5 wt% concentration represents an optimal balance, achieving maximum nanoparticle dispersion without significant aggregation, thereby enabling efficient stress transfer and synergistic piezoelectric enhancement between the BTO fillers and PVDF matrix. Various load resistances were connected with the specimens to characterize the output of the PVDF/BTO-5 composites. With an increase in load resistance, the voltage increased monotonously and almost did not change when the load resistance was up to 1000 MΩ (open circuit). For the current density, it decreased monotonously with increasing load resistance. Thus, the peak power density (27.5 nW cm^−2^) is obtained at the optimal load resistance of 500 MΩ. We calculate the power density by multiplying the voltage and current at the same load resistance. Note that the power density is obtained by controlling the stress at 10 N. The electrical performance is closely correlated with the loaded stress.

**Figure 5:**
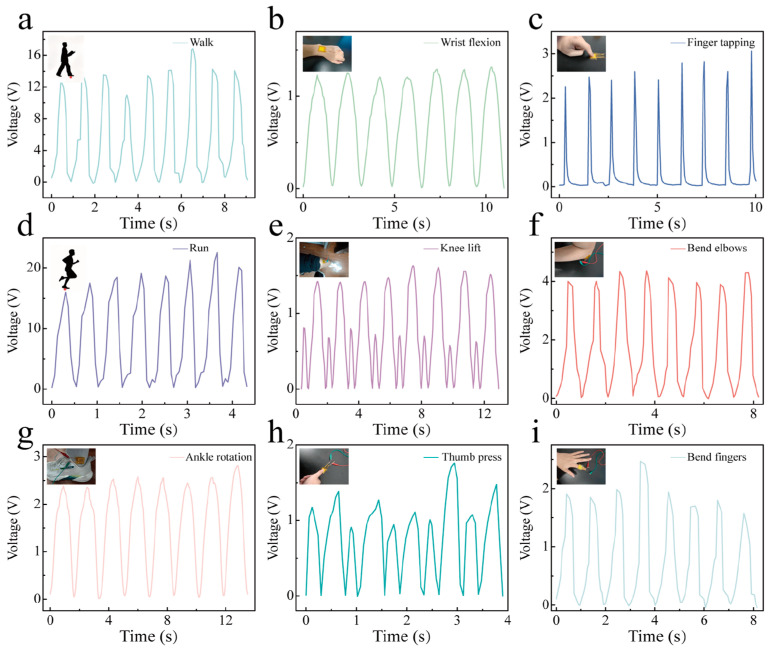
Output voltage of PVDF/BTO-5 composite with various human actions. (**a**) Walking; (**b**) wrist flexion; (**c**) finger tapping; (**d**) running; (**e**) lifting knees; (**f**) bending elbows; (**g**) ankle rotation; (**h**) pressing thumbs; (**i**) bending fingers.

The energy-harvesting and sensing capability is illustrated by the various human actions in Figure 5, such as walking, wrist flexion, finger tapping, etc. Due to the different stress imposed by distinct human actions, the output voltage is varied from 1 V to 20 V. The high output voltage of 20 V is comparable with the previous studies [34,35]. The angle of wrist flexion is around 10°, and the angle of the bending of elbows is around 70°. The output voltage is closely correlated with the amplitude of human motion [36,37]. Thus, the output performance could indicate the angles of various human flexions, which serve as sensing parameters for detecting various human motions.

When piezoelectric materials are subjected to pressure, they generate strain and an electric field. We employed a stress–electric field model to characterize the piezoelectric properties of the material, with the governing equations as follows:

T = c_E_S − e^T^E D = eS + ε_0_ε_rs_E (2)

where **T** denotes the stress tensor, **c^E^** represents the elastic stiffness coefficients, **S** is the strain tensor, **e** stands for the piezoelectric coupling coefficient, **E** is the applied electric field, and **D** indicates the electric displacement, while **ε_0_** and **ε_rs_** correspond to the vacuum permittivity and relative dielectric constant of the material, respectively. These parameters will be computationally fitted using COMSOL Multiphysics through the following workflow.

**Figure 6:**
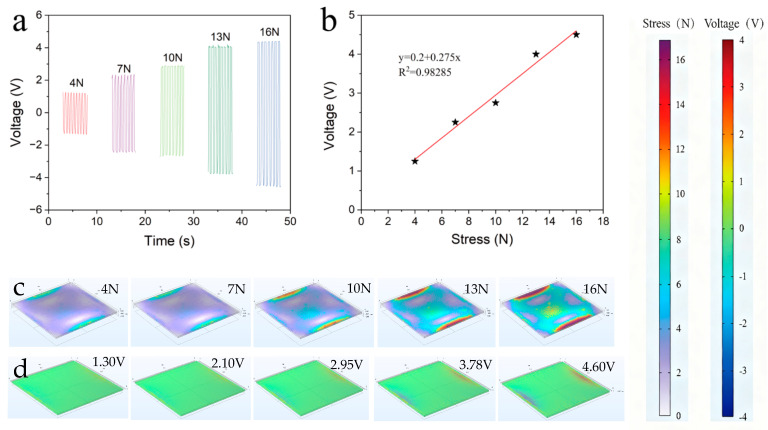
(**a**) Measured peak current under varying pressures; (**b**) voltage–pressure correlation characteristics; (**c**,**d**) deformation (**left**) and induced voltage (**right**) of the 5 wt% composite film under different pressures.

Voltage tests were conducted on the 5% concentration-doped film under different pressures. As shown in Figure 6a, the test results indicate that, as the pressure increases, the voltage generated by the film also increases. Under a 16 N force, an induced voltage of 4.5 V can be achieved, demonstrating that the film has good piezoelectric properties.

This section presents simulations of the different voltages and deformations produced when various forces act on the film. Figure 6 show the model established using COMSOL 5.2, employing a piezoelectric coupling model for piezoelectric fitting. The polarization direction is downward along the *z*-axis. For the PVDF modified at a 5% concentration, the measured dielectric constant is 10 at room temperature, and the modified PVDF has a density of 1.8 g/cm^3^, an elastic coefficient of 3.5 GPa, and a piezoelectric coefficient of 20 pC/N. The simulation of the modified PVDF under different forces shows the deformation and electric field generated by the material. It can be observed from the figures that, under different forces, the material undergoes certain deformations, which are within the acceptable range for flexible devices. Simultaneously, electric fields of varying magnitudes are generated, consistent with the measurement results in Figure 4, with a maximum voltage of 4.5 V. Equal but opposite induced voltages are produced at both ends, and the voltage increases with the applied force, aligning with the test results.

## 4. Conclusions

Self-powered pressure sensors based on the piezoelectric effect have received widespread attention and show great application prospects in flexible electronics and implanted devices. In this work, we designed and prepared a serious of high-performance pressure sensors by incorporating high-piezoelectricity lead-free BTO fillers in PVDF matrix. The performance is closely correlated with BTO content, and the maximum power density is up to 27.5 nW cm^−2^ at 10 N load stress. The output voltage is up to 20 V when the sensors are subjected to run motion, which is comparable with previous studies. The high energy-harvesting capability could be ascribed to the high *β* phase content (72.9%) and the introduction of BTO fillers. The fabricated piezoelectric sensor demonstrates outstanding overall performance, including high sensitivity (0.25 V N^−1^), a fast response time (~10 ns), and a low detection limit (0.01 N), making it highly suitable for monitoring subtle physiological signals and human motions. We investigated the energy-harvesting and sensing capability of the PVDF/BTO-5 composite across a series of human actions. The output voltage is closely correlated with the angles of flexion or load stress, thus serving as a foundation for precise pressure sensing. This work proposes a new strategy for designing high-performance self-powered piezoelectric pressure sensors, which will help to accelerate the development of flexible electronics and implantable devices.

## Data Availability

The original contributions presented in the study are included in the article, further inquiries can be directed to the corresponding author.
